# A comprehensive review on oleaginous bacteria: an alternative source for biodiesel production

**DOI:** 10.1186/s40643-022-00527-1

**Published:** 2022-04-22

**Authors:** Deepali Koreti, Anjali Kosre, Shailesh Kumar Jadhav, Nagendra Kumar Chandrawanshi

**Affiliations:** School of Studies in Biotechnology, Pt. Ravishankar Shukla University, Raipur, Chhattisgarh India

**Keywords:** Biodiesel, Bio-harvesting, Feedstock, Oleaginous microbes, Transesterification

## Abstract

Due to continuously increasing population, industrialization, and environmental pollution, lead to generating high energy demand which suitable for our environment. Biodiesel is an alternative renewable fuel source. According to the feedstock of production, biodiesel has been categorized into four generations. The main disadvantage of the first and second generation is the raw material processing cost that the challenge for its industrial-level production. Oleaginous bacteria that contain more than 20% lipid of their cellular biomass can be a good alternative and sustainable feedstock. Oleaginous bacteria used as feedstock have numerous advantages, such as their high growth rate, being easy to cultivate, utilizing various substrates for growth, genetic or metabolic modifications possible. In addition, some species of bacteria are capable of carbon dioxide sequestration. Therefore, oleaginous bacteria can be a significant resource for the upcoming generation’s biodiesel production. This review discusses the biochemistry of lipid accumulation, screening techniques, and lipid accumulation factors of oleaginous bacteria, in addition to the overall general biodiesel production process. This review also highlights the biotechnological approach for oleaginous bacteria strain improvement that can be future used for biodiesel production and the advantages of using general biodiesel in place of conventional fuel, along with the discussion about global policies and the prospect that promotes biodiesel production from oleaginous bacteria.

**Graphical Abstract:**

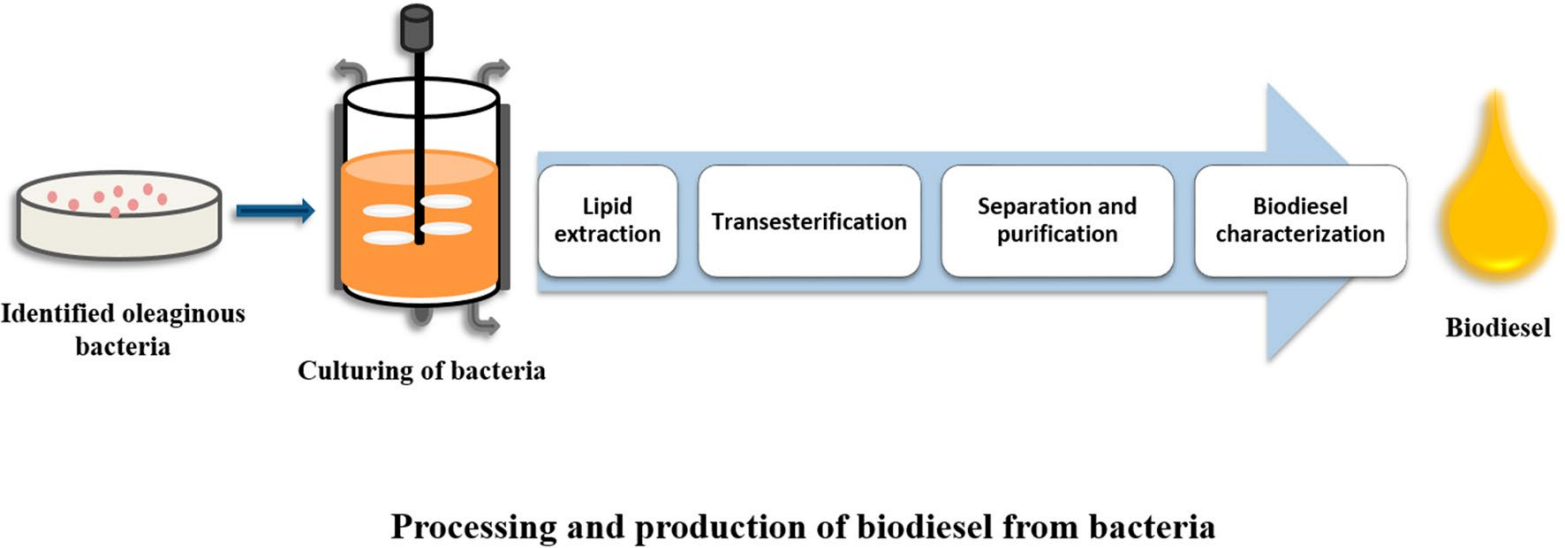

## Highlights

• Biodiesel from oleaginous bacteria and its importance are summarized.

• Biochemical pathways for fatty acid synthesis are described.

• Critical biotechnological approaches for bacterial strain improvement have been discussed.

• Sustainable biodiesel production, challenges, and future possibilities have been discussed.

## Introduction

Energy sources and their management is critical input for the economic development of any country. Currently, the energy crisis and continuously increasing energy demands are significant concerns for our society due to industrialization and the growing population (Enamala et al. 2018). Over the last several years, we have been dependent on fossil fuels for energy scenarios (Zhang et al. 2020a, b). Expected biofuel production for 2020 was to be 144 billion liters worldwide. In India, the crude oil price has been increased significantly in recent past years and reached a level of more than140 per barrel. Petro-based oil contributes about 95% of the requirement for transportation fuel, and the demand has been steadily rising (National policy on biofuel 2019). Developing countries such as India and China contribute significantly to this drastic increase in energy demand (Singh et al. 2020). International Energy outlook predicted that world energy consumption between 2016 to 2040 will increase 48% and require another carbon–neutral energy source to fulfill energy requirements (John et al. 2016). The transportation sector is the primary source of air pollution due to the excessive consumption of fossil fuels (Biofuels Annual 2019). Fossil fuels are fuels formed by natural processes, such as the anaerobic decomposition of buried dead organisms. The combustion of fossil fuel produces Methane (CH_4_) together with the emission of Carbon dioxide (CO_2_), Carbon mono oxide (CO), Nitrogen oxide (NO_X_), Sulfur oxide (SO_X_), and moisture. Among them, carbon emission from CO_2_ is a significant concern in mitigating global warming as it is rapidly increasing, and this gas is a significant contributor to the greenhouse effect (Vasudevan et al. 2008; Sankaran et al. 2019). According to the current research, approximately 98% of greenhouse gas emissions in the atmosphere result from fossil fuel combustion (Dahman et al. 2019; Pant et al. 2019). It is estimated that CO_2_ emissions will be worldwide risen from 32.2 billion metric tons to 35.6 billion metric tons in 2012 to 2020 and 43.2 billion metric tons in 2040 (John et al. 2016). According to the Intergovernmental Panel on Climate Change (IPCC), approximately 69% of CO_2_ emissions are from the energy sector, including 14% from the transportation sector only (Dahman et al. 2019). Because fossil fuel resources are limited, non-renewable, and polluting by nature, there is a need for alternative fuel that is more suitable for the and agricultural waste environment. On the other hand, bioenergy or biofuels are renewable, environment-friendly and technically feasible; therefore, this can be the best alternative energy source for the upcoming generation.

Biodiesel is one of the most prominent renewable energy resources. It is a methyl or ethyl ester formed by transesterification of fatty acids, such as oil derived from plants, animals, organic waste, and microorganisms (Mahlia et al. 2020; Patel et al. 2020a). Biodiesel contains more O_2_ than petroleum diesel and has higher combustion efficiency with lower sulfur and aromatic content. They also have a higher flash point, higher cetane number, and are biodegradable. According to the feedstock of production, biodiesel was categorized into four generations (Alawana et al. 2019; Aron et al. 2020; Singh et al. 2020). Biodiesel produced from various foods stock sources such as edible plant oil and animal fat is called first-generation biodiesel. Second-generation biodiesel is produced from non-edible feedstock, such as the non-edible oil, the seed of *Jatropha*, food waste, animal fat waste, and agricultural waste (Ferrero et at. 2020; Srinivasan et al. 2020) etc. When plants (from the second generation) are used for biodiesel production, approximately 70–80% of the whole production cost is associated with its harvesting and processing (Adhikaria et al. 2021). Biodiesel produced from microbes is categorized in third-generation biofuels (Allen et al. 2018; Hossain and Mahlia 2020; Zhang et al. 2020a, b; Zeghloulia et al. 2021). The fourth-generation was recently introduced in which photobiological solar fuels, electro-fuels and synthetic cells are used for biodiesel production (Singh et al. 2020). Table 1 describes the sources, benefits and challenges of different generations of biodiesel. Yeast and bacteria utilize a reduced carbon source for their energy needs; these strains can metabolize various carbohydrates, such as residual sugars (for example, molasses or lactose). An extensive amount of low-cost carbon-rich sources is available to produce biodiesel through fermentation by microorganisms without competing with food production. Microorganisms that contain more than 20% of microbial lipid is called oleaginous microorganism, and it is considered alternative feedstock for the production of oil and fat (Maa et al. 2018). Biodiesel production using microbial lipids, called single-cell oils, has engrossed great attention globally (Carsanba et al. 2018). Oleaginous microorganisms mainly synthesize lipids with 4 to 28 unbranched carbon chain lengths (Patel et al. 2020b), and they can be saturated or unsaturated fatty acids depending on the nature of the carbonated hydro chain and the number of the double bond.

**Table 1:** Source, benefits and challenges of different generations biodiesel (Leong et al., 2018; Sigh et al., 2020)

S. N	Biofuels	First generation	Second generation	Third generation	Fourth generation
1	Source	Edible oil feedstock-like palm oil, soybean, rapeseed, sunflower & corn, etc	Non-edible feedstock-like seeds of jatropha, food waste, animal fat waste and agricultural waste, etc	Oleaginous microbes, such as bacteria, fungus, Yeast microalgae etc	Photobiological solar fuels, electro-fuels and synthetic cells
2	Benefit	Renewable source, biodegradable and their biodiesel conversion process is easy	Renewable, biodegradable, not compete with food crops	Renewable, biodegradable, not compete with food crops, the land is not required, no dependency on climate conditions and a higher growth rate	Renewable, Production rate is higher, High energy content, inexhaustible
3	Challenge	Requires land, manpower for cultivation and rising food price	Requires extra land and manpower for cultivation and production cost is high	Production low for commercialization and difficult to maintenance etc	Research is on infancy level

Many microorganisms produces hydrocarbons as metabolic by-products from fatty acids and triacylglycerol (TAG). TAG is used as an energy reserve by eukaryotic organisms, such as yeast, fungi, plants, and animals. However, it has not much been explored in the case of bacteria (Bharti et al. 2014a). TAG biosynthesis by bacteria groups is achieved in a high concentration of carbon sources, such as sugars, organic acids, alcohols, n-alkanes, branched alkanes, phenylalkanes, oils, and coal (Kumar et al. 2016). Bharti et al. (2014a) and Kumar et al. (2020) reported that the microbial fatty acid and TAG synthesized by bacteria could use as a starting material of microbial lipids source for biodiesel production. The production of this microbial lipids has numerous advantages, such as less laborious, less time-consuming process, less affected by season and climate, and easier to scale up than second-generation feedstock. Some bacterial species, including *Streptomyces, Rhodococcus, Arthrobacter, Acinetobacter, Mycobacterium, Bacillus,* and *Nocardia,* have been reported with their high lipid content (Carsanba et al. 2018; Maa et al. 2018), in which *Rhodococcus* strain are the most studied bacterial species with an abundance of more than 70% of lipid of their dry weight.

Bio-lipids from oleaginous microorganisms also have an advantage over vegetable oil in fatty acid composition and fatty acids composition can modify to the desired level by changing the source of nutrients or substrates and metabolic engineering strategy. It also reported that oleaginous bacteria efficiently use high-carbon waste for lipid production (Kumar et al. 2015; Kot et al. 2017). Using this high-carbon waste, such as sewage sludge (Ceaet al. 2015), and food waste, the overall production cost reduced up to 45%. Some chemolithotrophic bacteria use CO_2_ as a carbon source and produce a significant amount of extracellular lipids, which will utilize for biodiesel production along with CO_2_ mitigation. Bacterial strains such as *Serratia* species*, Sulfo bacillus* and *Oscillochloris* previously reported fixed atmospheric CO_2_ through the Calvin cycle. They synthesized various value-added products, including lipids exopolysaccharides, bioplastics, and fatty acids (Bharti et al. 2014a; Kumar et al. 2015; Maheshwari et al. 2018). Along with several advantages, there are some challenges associated with oleaginous bacteria, such as identification and isolation difficulty to maintain a pure culture and commercialization.

**Fig. 1:**
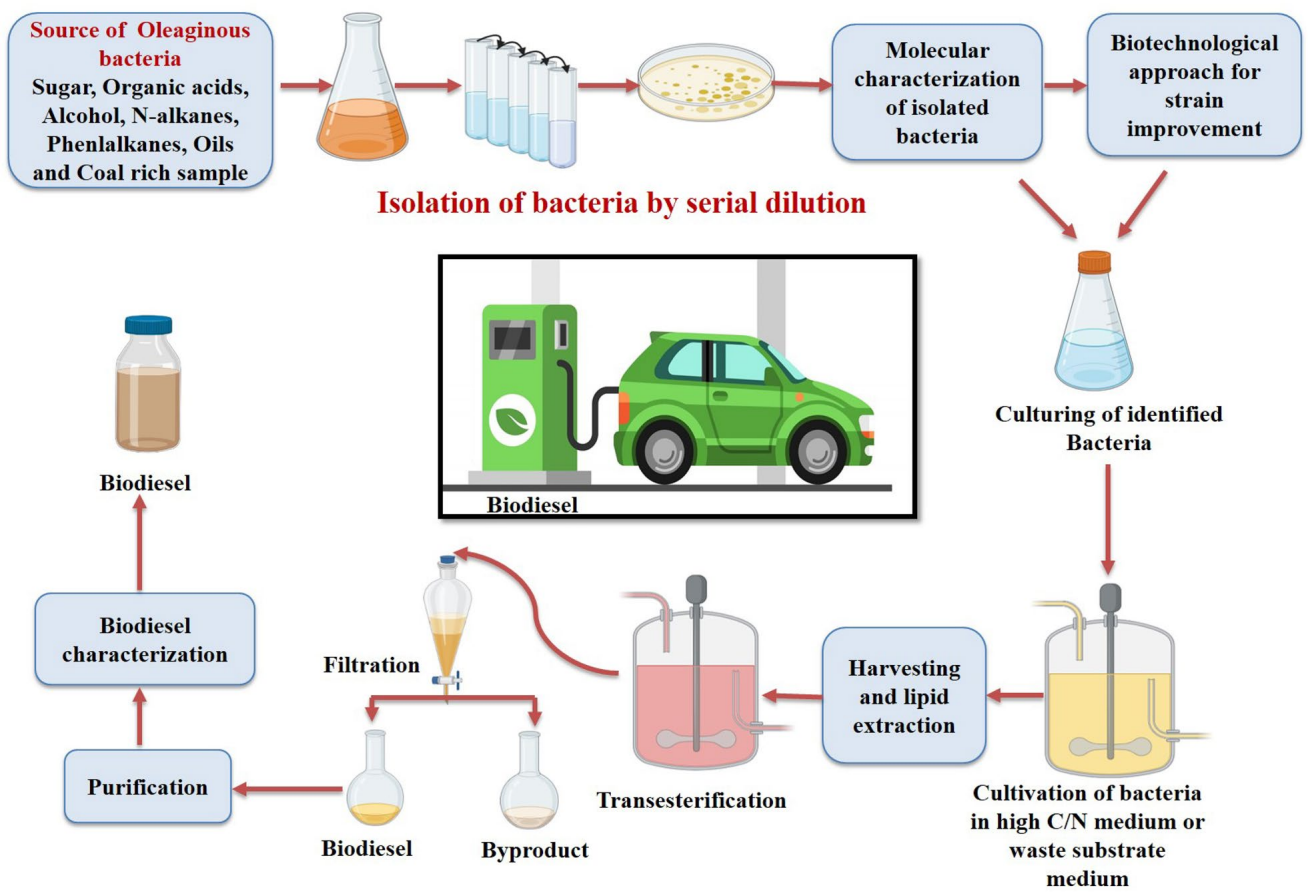
Flow chart biodiesel production from oleaginous bacteria

Excellent reviews and research articles are available for various biofuel sources and their production on the international database (Banerjee et al. 2017; Enamala et al. 2018; Singh et al. 2019; Arous et al. 2019; Kumar et al. 2020; Wang et al. 2021). This review covers the biochemical pathways of lipid accumulation, the process of biodiesel production by bacteria, and all the possible aspects for future biodiesel production enhancement strategies that can apply in bacterial biodiesel production. The findings of the present review can help understand the importance of oleaginous bacteria and their further application in biodiesel production as well their industrial implementation. The summarized biodiesel development process from oleaginous bacteria is schematically shown in Fig. 1.

## Lipid/fatty acid content reported in different types of bacteria

The bacteria cell growth rate is high, but lipid accumulation in bacteria is low compared to fungus and microalgae. Bacterial lipids are produced in tiny droplets in bacterial cytosol with high cell growth rates under simple cultivation methods, and some strains accumulate oil under a particular environment (Qadeer et al. 2017). Poly hydroxyl alkanoic acids are the most abundant class of neutral lipids in most bacterial species and serve as intracellular carbon and energy storage compounds (Carsanba et al. 2018). TAG accumulation has been reported in some cases (Bharti et al. 2014a). It reported that the accumulation of lipid in a bacterial culture mainly occurs during the stationary growth phase. Microbial lipids differ by their composition and contents, depending upon the biochemical synthesis pathways. The microbes can follow different factors that affect the lipids synthesis pathways, such as culture conditions, carbon sources, temperature, pH, and nutrient availability. *Rhodococcus, Mycobacterium, Streptomyces, Nocardia,* and *Acinetobacter* are some bacterial genera that produce triacylglycerols in the maximum amount. Gram-positive bacteria such as *Rhodococcus opacus* and *Arthrobacter* species are reported for high biomass and storing fatty acids up to 87% of their cellular dry weight (CDW). These fatty acids and their derivatives act as precursors for synthesizing their cell envelopes (Kumar et al. 2020). *R. opacus* accumulated TAG up to 86% of its cellular dry weight, and it was the most studied oleaginous bacterial strain subject to pilot-scale fermentation and optimization. It was reported that the *Clostridium* synthesize and store hydrocarbon ranging from C11 to C35 with the majority of medium-chain n-alkanes (C18–C27) and higher range n-alkanes (C25–C35). *Vibrio furnissii* bacterium belongs to the halotolerant microbes, which synthesizes and produces intracellular and extracellular hydrocarbons ranging between C15 and C24, having similar properties to kerosene and light oil. Gram-negative bacterial strains have not been much reported for TAG accumulation compared to gram-positive bacteria (Babu et al. 2020; Kumar et al. 2020). Some gram-negative bacterial strains such as *Alcanivorax or Marinobacter* species are capable of synthesizing wax ester and a small extent of TAG. These quantities increase when these bacterial strains are grown on specific carbon sources (n-alkanes or olive oil). A gram-negative strain *Aeromonas*, content fatty acid up to 12% CDW in which 30% is eicosapentaenoic acid (polyunsaturated fatty acid). Genus *Nitratireductor* (*α-proteobacterium*), oleaginous gram-negative bacterial strains, have been reported for wastewater treatment application using short-chain organic acids as carbon sources. This bacterial strain also produces a mixture of fatty acids have TAG, squalene, and methyl ester of 2-butenoic acid. Recently, the genetic engineering tool is the most potential technique for optimizing and selecting carbon sources for of lipid and fatty acids production (Rottig and Steinbuchel 2015).

## Biochemical pathways for fatty acid synthesis in bacteria

Lipids are an essential and significant structural component of the cell, and cells make different lipids for different functions. Fatty acids are the components of the lipid and fatty acids are precursors for various biomolecules, such as phospholipids, sphingolipids, sterols as secondary metabolites and signaling molecules (Campbell and Cronan 2010; Janben and Steinbuchel 2014). Bacterial fatty acids are similar to the most abundant species in eukaryotic cells, but bacterial fatty acids are shorter than eukaryotic fatty acids. They generally lack polyunsaturation, and the monoenoic C18 acids have different double-bond positions. Some bacteria make branched-chain fatty acids, whereas others make 3-hydroxy acyl acids. In general, fatty acid synthesis (FAS) pathways are present in two distinct molecular forms called type I and type II. FAS catalyzes by a single, large polypeptide unit composed of several distinct domains in the type I system, which is present in mammals (including humans).

**Fig. 2:**
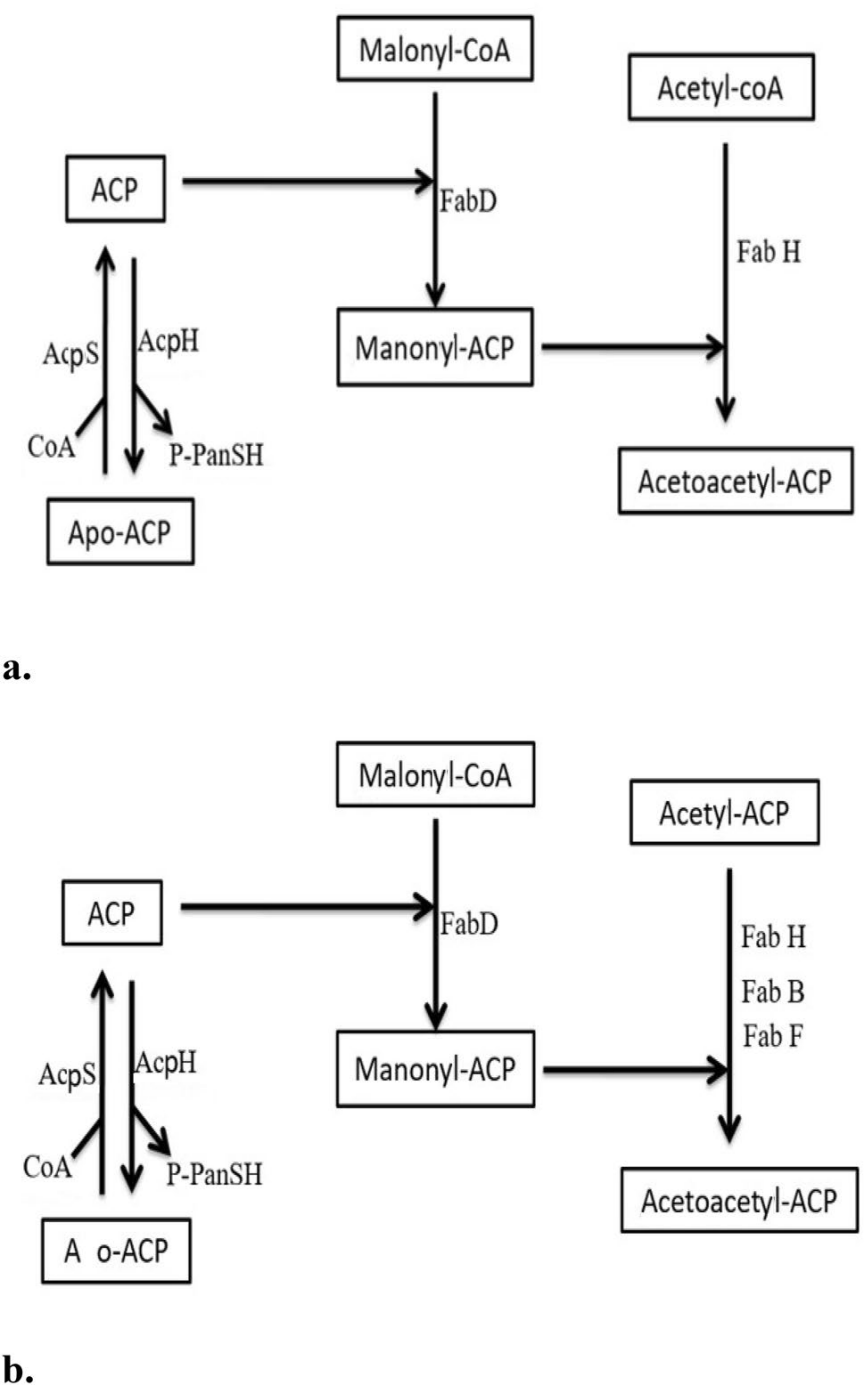
Initiation steps (**a** & **b**) in the type II fatty acid synthesis pathway of *E.coli*

**Fig. 3:**
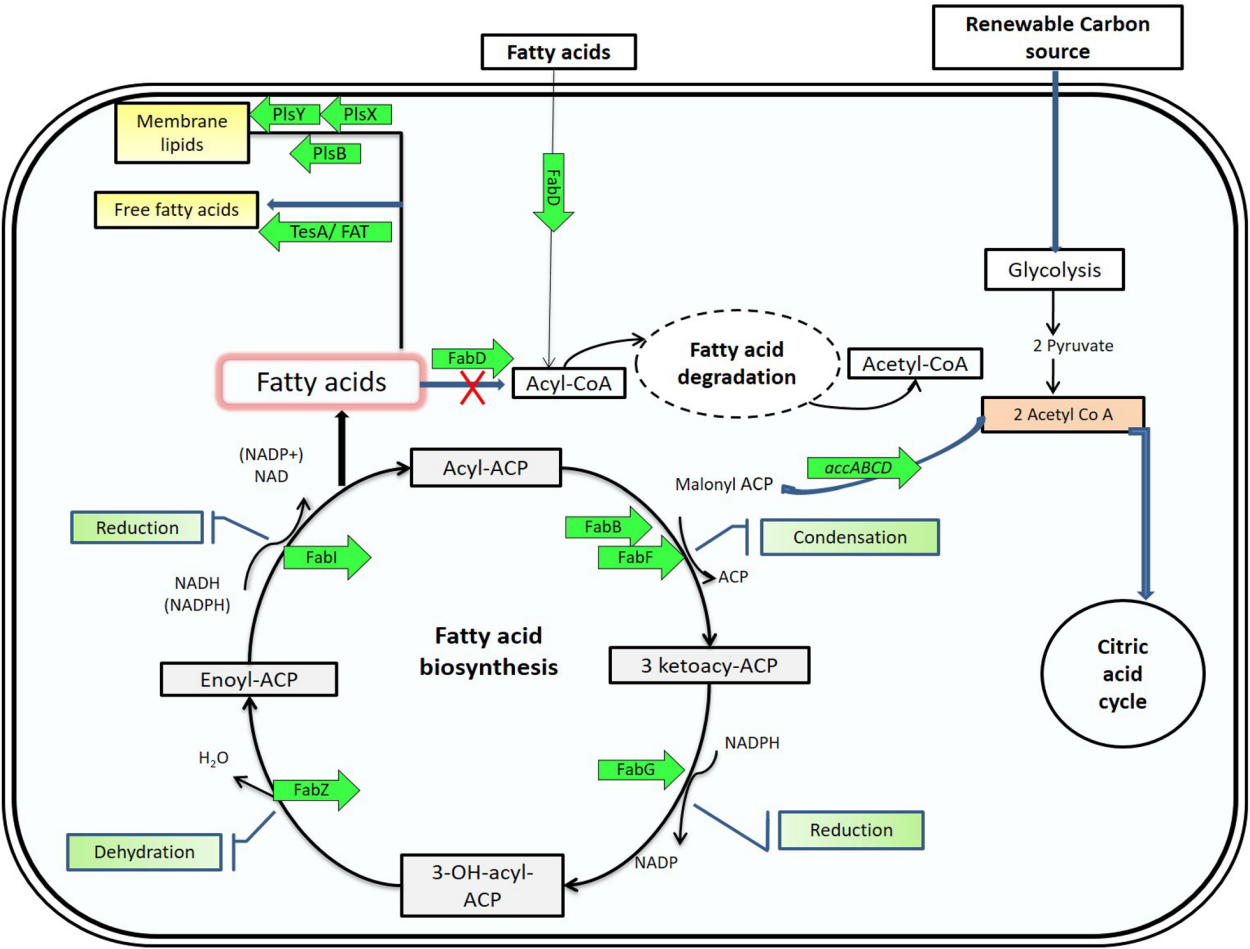
Fatty acid synthesis and degradation in *E.coli.* Green arrow indicates gene that code for particular protein or enzyme required for the reaction. Red Cross indicate blocking particular pathway for inhibit fatty acid degradation

In the type II system (present in bacteria, plants and protozoa) (Ratledge et al. 2004; Qadeer et al. 2017), having components, including the acyl carrier protein (ACP), exist as discrete proteins. The two FAS systems (I and II) and related activities belong in structure and function, but they generally lack sequence homology. Precursors are derived from the acetyl-CoA in these fatty acid biosynthesis pathways. Acetyl-CoA is the acetyl transfer agent, ubiquitous by nature, plays a role in central metabolic pathways, and serves as an intermediate for fatty acid synthesis (Wang et al. 2021). Fatty acid synthesis in bacteria involves initiation and elongation steps, as shown in Figs. 2 and 3. An Acyl carrier protein (ACP) is a slight, very acidic, and highly soluble protein in this fatty acid synthesis pathway. All the intermediates of this fatty acid synthesis pathway are covalently bound to this ACP. Table 2 demonstrates detailed information on enzymes that evolve in lipid biosynthesis.

**Table 2:** Enzymes and genes envolve in lipid biosynthesis (Campbell and Cronan, 2010; Wang et al. 2021)

S. N	Name of protein/ enzyme	Protein size (active form)	Gene name	Functions
1	Acetyl-CoA *Carboxylase(ACC)* 1.*accA Carboxyl-* *transferase subunit* 2.*accB Biotin carboxyl* *carrier protein(BCCP)* 3.*Biotin carboxylase* 4.*Carboxyl-transferase subunit*	35 kDa (× 2 with 2AccD) 16.7 kDa (+ biotin × 2) 49.3 kDa(× 2) 33.2 kDa(x2with2 AccA)	*accA* *accB* *accC* *accD*	Conversion of acetyl-CoA to malonyl-CoA
2	Fab D/malonyl-CoA: ACP *transacylase*	32.3 kDa (× 1)	*fabD gene*	Conversion of malonyl-CoA to malonyl-ACP
3	FabH /3-ketoacyl-ACP *synthase* III also called acetoacetyl-ACP *synthase*	33.5 kDa (× 2)	*FabH*	Catalyze 3- ketoacyl-ACP synthase reactions
4	FabB /3-Ketoacyl- ACP/*synthase* I	42.6 kDa (× 2)	*FabB*	Catalyze 3- ketoacyl-ACP synthase reactions
5	FabF /3-Ketoacyl-ACP *synthase* II	43.0 kDa (× 2)	*FabF*	Catalyze 3- ketoacyl-ACP synthase reactions
6	FabG/ an NADPH-dependent 3-ketoacyl-ACP *reductase*	25.6 kDa (× 4)	*FabG*	Conversion of 3ketoacyl-ACP to 3OH acyl ACP
7	FabZ / 3-hydroxy acyl-ACP *Dehydratase*	16.9 kDa (× 2)	*FabZ*	Removes H_2_O from 3OH acyl ACP and forms enoyl—ACP
8	FabI / an enoyl-ACP reductase	27.7 kDa (× 4)	*FabI*	Reduction of enoyl ACP to acyl ACP

### Initiation

The initiation reactions provide the substrates for the first chain elongation step. Fatty acid synthesis primed by acceptor C2 unit short acyl-Coenzyme A (acyl-CoA) thioesters, incorporating specific primers units, determines whether the final acyl chain will be even or odd chain will be straight or branched. In *E. coli,* straight-chain fatty acids are primed by condensing acetyl-CoA with malonyl-ACP to yield acetoacetyl-ACP, CoA, and CO_2_. According to malonyl-CoA is required for the entire elongation step for fatty acid biosynthesis, and it formed in the first step. This reaction is catalyzed by acetyl-CoA carboxylase (ACC). Malonyl-CoA was utilized in fatty acid biosynthesis only in malonyl-ACP and this conversion was catalyzed by ACP transacylase (Fab D). The condensations of acetyl-CoA with malonyl-ACP were catalyzed by ketoacyl-acyl carrier protein synthase III (FabH). Similarly, straight-chain fatty acids are synthesized when synthesis is primed bypropionyl-CoA (Pfleger et al. 2015). There are some mechanisms or pathways for initiating fatty acid biosynthesis in *E. coli* (Qadeer et al. 2017). First, acetyl-acetyl-CoA condenses to yield acetoacetyl-ACP catalyzes by FabH (3-ketoacyl-ACP synthase III), as revealed in Fig. 2a, b. In the second pathway, the acetate moiety was first transferred from acetyl-CoA to acetyl-ACP by the transacylase activity of FabH. The acetyl-ACP was then condensed with malonyl-ACP by FabB (synthase I) or by FabF (synthase II) shown in Fig. 2a, b.

### Elongation

#### Claisen condensation

In the fatty acid synthesis cycle, the reaction was Claisen condensation of an acyl-enzyme/acyl thioester (acyl-ACP or for FabH, acetyl-CoA) with malonyl-ACP to form a 3-ketoacyl-ACP, CO_2_, ACP (or CoA) and free enzyme. In *E. coli,* three enzymes catalyzed 3-ketoacyl-ACP synthase reactions, and these enzymes were referred to as synthases I, II, and III, but more recently called FabB, FabF, and FabH, respectively, after their gene names. Enzymes FabB and FabF have a dimeric protein structure and can participate in the saturated and unsaturated fatty acid synthesis reactions. It is the irreversible step in the elongation cycle of fatty acid synthesis, and thus 3-ketoacyl-ACP synthases play a crucial role in regulating the product distribution of this pathway (Janben and Steinbuchel 2014).

#### Reduction

The 3-keto-thioester (3-ketoacy-ACP) is reduced by NADPH-dependent 3-ketoacyl-ACP reductase (Fab G) and forms 3-hydroxy acyl- ACP. In *E. coli,* only a single NADPH-specific 3-ketoacyl-ACP reductase is present and functional with all acyl chain lengths (Javidpour et al. 2014).

#### Dehydration

Removal of a water molecule from the three hydroxy acyl-ACP is catalyzed by 3-hydroxy acyl-ACP dehydratase (FabZ) and form Enoyl-ACP. Dehydratase efficiently catalyzed the dehydration of chain 3-hydroxy acyl-ACPs and a long chain of saturated and unsaturated 3-hydroxy acyl-ACP.

#### Reduction

An enoyl-ACP reductase (FabI) reduction process has been catalyzed and gives an acyl-ACP as an end product. Enoyl-ACP reductase was the last enzyme of the fatty acid cycle. FabI pulls the other reversible cycle steps by controlling other enzymes activity (FabG and FabZ) for accurate fatty acid biosynthesis. Acyl-ACP can serve as a substrate for another round of elongation or if sufficient chain length synthesizes. It can transfer into complex lipids. The trans-3-decanoyl-ACP FabI is followed by elongation by either FabB or FabF to form palmitic acid, the primary saturated fatty acid in *E. coli.*

## Factors affecting lipid synthesis and accumulation in bacteria

### Carbon (C) and Nitrogen (N) source

Carbon and Nitrogen are essential sources for microorganism growth and affect cellular lipid accumulation. An appropriate C/N percentage triggers lipid accumulation. Usually, a high C/N ratio in the medium increases the total lipid yield (Qadeer et al. 2017). Excess carbon source and limiting nitrogen in providing growth medium stimulates the synthesis of triglycerides, because cellular growth is impaired, and the cell utilizes the carbon source for neutral lipid biosynthesis (Bharti et al. 2014b; Kumar et al. 2020). The biosynthesis of fatty acids and their composition in the cells depends on the available carbon source and C/N ratio. Kumar and Thakur (2018) reported produced biodiesel production from *Serratia* species lipid using sodium bicarbonate and glucose (carbon source) enriched culture medium. Recombinant *E. coli* in fermentation produced 4.8 and 3.8 g/L FFA using glycerol or glucose with woody biomass hydrolysate as a carbon source. Behera et al. (2019) isolated high lipid accumulating bacteria from dairy waste water. They reported high lipid accumulation (80–90%) using different cellulose, lactose, sucrose and starch, in which lactose supplemented culture gives the highest accumulation (90%) and 1.2 g/l lipid productivity. However, raw glucose and other pure compounds are not cost-effective for large-scale biodiesel production. Future research is in progress to optimize effective and cheap carbon sources for significant lipid and biodiesel production from bacteria.

### Other nutrients

Other nutrients such as Ammonium chloride, Sodium nitrate, Phosphate, Magnesium sulfate have also controlled the accumulation of lipids in the bacterial cell in nutrient deficiency conditions. Increasing a 10% concentration of Ammonium chloride in the culture medium could increase the lipid content (Sitepu et al. 2014). Increasing the engagement of NaNO_3_ in the culture media could decrease lipid accumulation in *Cyanobacteria* cell biomass. Sulfate, Magnesium Sulfate, and Phosphate deficiency can also cause the accumulation of lipids in certain oleaginous microbes (Kumar et al. 2020). According to Arora et al. (2016) N/P limitation could enhance lipid production. Nayan et al. (2020) said increased lipid concentration in a microorganism (*Chlorella kessleri*) in Phosphorus limited growth. Notably, not much work done in nutrients affects oleaginous bacteria compared to other organisms, such as algae and yeast.

### Temperature

Temperature is also an essential factor for lipid accumulation in microorganisms. It affects the degree of saturation in the triacylglycerols production, because different optimum temperatures require particular enzyme activity to evolve in lipid metabolism. Mostly 30 to 36ºC temperatures are preferred by cultivating oleaginous bacteria for biodiesel production (Kumar et al. 2017a, b; Kumar and Thakur 2018).

### pH

pH value and the carbon present in the culture medium also affect lipid accumulation. In general, a pH ranging from 5.0 to 6.5 is most suitable for lipid accumulation in oleaginous bacteria (Kumar et al. 2017a, b). Kosa et al. (2013) reported a positive influence with an increase in pH in *R. opacus.* A research study and review said that (Kot et al. 2017; Qadeer et al. 2017) in some bacterial species shows that pH does not significantly affect lipid accumulation, but it can affect the overall biomass production rate. Thus, more research will require for the assessment of pH effect on lipid accumulation and other essential compounds linked with lipid accumulation.

## Biodiesel production process from oleaginous bacteria

### Selection of oleaginous bacterial strain

Depending on the growth condition of microorganisms, it has specific or different lipid accumulation capacity even in the same microorganism. Therefore, screening is required for determining the lipid composition in a microorganism or bacteria (Behera et al. 2019). The initial screening of the oleaginous bacteria tested for their ability to grow on a nitrogen-deficient medium (Bharti et al. 2014b). An isolated bacterium produced on a nitrogen-limited medium was further screened based on its capacity to store lipids in CDW. The selected isolates were grown for a particular culture condition, and when the stationary phase reached, the cells harvested by centrifugation and lipid contents were estimated. The cell pellets dried to constant dry weight. Finally, the CDW was measured and further analyzed for lipid accumulation capacity by the gravimetric method. Some other screening methods used, such as the Fat stain method, Soxhlet extraction method, GC extraction method, near-infrared reflectance spectroscopy (NIRS) method, neutral (trans esterifiable) and polar lipids content determined using flow cytometry or nile red strain method (Behera et al. 2019; Zhang et al. 2020a, b).

### Culturing /cultivation of bacteria for biomass production

Culturing selected bacteria used in biodiesel production is crucial, affecting the overall production cost. The implement biodiesel production from bacteria, there is a need to search for a cheap and cost-effective method. There are many substrates used for bacteria cultivation. Bioaccumulation of lipids is mainly dependent on the substrate used by the microorganism. A high C/N ratio is required for lipid biosynthesis, and glucose is a frequently used substrate for high lipid accumulation. Sometimes xylose was also used as a substrate. However, using a pure form of these substrates increases total biodiesel production cost, because the more significant part (about 85%) of the cost depends on feedstock, and commercialization is not economically suitable. For commercialization, bacterial feedstock substrate should be low cost and high availability by nature. The economic viability of microbial oils remains uncertain due to the high price of cultivation substrate, which accounts for 40–80% of overall biodiesel production cost (Cho et al. 2015). Therefore, the oleaginous microorganisms are cultivated on low-cost substrates to produce economically sustainable microbial oil. Instead of glucose, nutrient-rich wastewaters (low-cost substrates) can be a good alternate for oleaginous microorganism’s cultivation to develop an environmentally and economically viable biodiesel production process (Arous et al. 2019). A different type of waste (food, agricultural waste, sewage sludge) rich in organic nutrients is considered a good substrate source for bacterial growth and lipid accumulation (Cea et al. 2015). The following section describes some alternative substrates that are used in bacteria cultivation.

### Dairy wastewater

The dairy industry generates large volumes of wastewater with a high concentration of fats and oils. The dairy wastewater is efficiently utilized to grow the lipid accumulating microorganisms for nutrient purposes (Kumar et al. 2015). Behera et al. (2019) isolated rod-shaped oleaginous bacterium DS-7 from dairy wastewater and investigated their lipids accumulation potential. This bacterium efficiently utilizes dairy wastewater by approximately 50% BOD reduction and accumulates lipid up to 72%. Few studies reported the ability of oleaginous bacterial stain to treat wastewaters with simultaneous lipid production. *R. opacus* was used to treat and valorize dairy wastewater and produced microbial lipid (Kumar et al. 2015). Bacteria accumulated up to 14.28% w/w lipid and reduced the initial COD by 30% from raw dairy wastewater. The lipid content and COD removal efficiency increased up to 33% and 62% when added mineral salts were to the dairy wastewater. *R. opacus* PD630 reports efficient dairy wastewater treatment with high lipid accumulation (Gupta et al. 2017). This strain (*R. opacus*) was studied for a successful scale-up process using a bioreactor for cultivation. In this experiment, three different culture modes were applied: fed-batch, continuous and constant cell recycling, and found that the continuous cell recycling manner of cultivation showed a maximum lipid accumulation (3.4 g /L).

### Municipal sewage sludge

Sewage sludge is an organic waste by-product generated by the wastewater treatment plant's primary and secondary treatment process (Sharholy et al. 2007; Zhou et al. 2020). Municipal sewage sludge (MSS) from municipal wastewater treatment plants might be an alternative feedstock for oleaginous bacteria cultivation and biodiesel production (Karn and Kumar 2019; Yang et al. 2020). It has a variety of different elements, such as triglycerides, diglycerides, monoglycerides, phospholipids, free fatty acids, and organic nutrients. Thus the reason is making an ideal feedstock for the production of biodiesel. Different types of wastewater sludge (primary, secondary, blended, and stabilized sludge) are the source of other bacteria that are oleaginous by nature. Kumar and Thakur (2018) experimented and deliberated on biodiesel production (yield-11.21 ± 0.19%w/w, with balance FAMES composition) from chemolithotrophic, oleaginous bacterium *Serratia* species by utilizing MSS media as a carbon source for the growth of bacteria. They also analyzed the 5% blends and found better fuel properties, which will employ without modifying the existing engine. Jamal et al. (2019) worked on wastewater treatment, extracted lipid from wastewater sludge, and produced biodiesel. Lipid extraction using a mixture of polar and nonpolar solvents and sulfuric acid used for transesterification. Moreover, a 78% ester yield from 2 h of ex-situ esterification at 7% catalyst concentration.

### Agricultural waste and lignocellulosic biomass

Lignocellulosic refers to the dry matter of the plant. Lignocellulosic biomass is the forest and agricultural residue and commercial energy crops. It represents the most abundant natural resource that will be utilized to produce biofuels. Lignocellulose consists of three polymers: cellulose 35–55%, hemicellulose 20–40%, and lignin 10–25%. Lignocellulosic biomass rich in sugars can help the growth of heterotrophic organisms (Cheah et al. 2020). The oleaginous microorganism can utilize cellulose-based sugars grown on lignocellulosic hydrolysates biomass. In particular, nitrogen-deficient conditions produced lipid up to 70% of DCW (Kumar et al. 2017a, b). Many researchers have reported the fermentation of lignocellulosic biomass to biogas or ethanol (Sun et al. 2021). Certain studies have explored the possibility of producing TAGs from lignocellulosic biomass for biodiesel production.

### Food waste

The various food waste such as potato infusion, vegetable, and fruit wastes use as a substrate for bacteria cultivation and lipid production. *R. opacus* was grown on orange waste, apple, pomace, or sweet whey waste, found high lipid content of about 85% (w/w of CDM) and when *Gordonia* species DG cultivated on potato infusion, orange waste, carob waste, and apple pomace then achieved 70% (w/w of CDM). Table 3 shows different forms of waste having additional potential with microorganisms for lipid accumulation.

**Table 3:** List of various substrates for growth of bacteria and their lipid content (Kumar et al. 2015; Kumar et al. 2017a, b; Goswami et al. 2017)

S.N	Bacterial Species	Substrate for growth	Biomass concentration (g/L)	Lipid content of dry cell weight (DCW) %
4	*Rhodococcus opacus*	Dairy wastewater with mineral salt medium	6.6	33.30
5	*R. opacus*	Orange waste	ND	85.00
6	*Rhodococcus* species	Lignin compounds	ND	4.08
7	*R. opacus*	Oxygen-pretreated kraft lignin (discharged from the pulp and paper industry)	0.5	14.20
8	*R. opacus*	Toxic biomass gasification wastewater	0.7	54.30
9	*Gordonia* species	Sugar cane molasses	ND	96.00

Remark—ND: not detected

### Lipid extraction and lipid characterization

Lipid extraction has contributed approx. 30–40% of the total biodiesel production. The efficiency of lipid extraction and lipid recovery is directly influenced by the use of microorganism (Khoo et al. 2020). Thus, sophisticated processes were optimized for lipid extraction, incorporating various steps including different extraction procedures such as solvents extraction, Soxhlet method, Folch method, Bligh and Dyer method, Supercritical fluids, and ultrasonication, etc. for achieving quality products. Chloroform, Methanol, and n-Hexane are commonly used solvents in all extraction methods.

Ultrasonication is a new technique that is broadly utilized to enhanced bio-product production from various organic wastes. It reported that ultrasonication applies to scale-up, and it has been used in many processes such as biogas production and crude oil recovery (Wang and Gu 2017). Chen et al. (2018) assessed the application of ultrasonication in biodiesel production and overcame the difficulties in the production process from oleaginous microorganisms. The oleaginous microbe is accessible and easy to handle to cultivate and characterize chemical reactions in the cell. Moreover, high efficiency of lipid extraction, transesterification, and in-situ transesterification methods have successfully been used for biodiesel production. Furthermore, this technique gives higher biodiesel yield with no extra energy and cost investment. There are many advantages such as sort reaction time low temperature. Ho et al. (2016) and Sivaramakrishnan and Incharoensakdi et al. (2017) reported that ultrasonication could increase the bioavailability of organic waste by disrupting the complex and large molecules to simple forms. According to Han et al. (2016), ultrasonication can stimulate autotrophic oleaginous microbes (algae) for lipid production. By affecting the integrity of cell walls, rupturing the linkages between the phospholipids molecules, and disrupting the cells (Abdullah et al. 2016), ultrasonication is utilized for lipid extraction from microalgae (Wang et al. 2016; Chong et al. 2021). In-situ trans-esterification is the new way of biodiesel production in which oil located in microbes can directly convert the biodiesel without disturbing the biodiesel profile (Chen et al. 2018). Many scientists accounted for the application of ultrasonication in both esterification and transesterification. Apart from lipid extraction, various pretreatment methods enhance lipid recovery. Physical, chemical, and enzymatic methods are used in the series to pretreat extracted lipids (Patel et al. 2020b). Lipid extraction methods are well established and standardized in plant and algae-based biodiesel production (Mat Aron et al. 2021). Conventional methods are easy, economic and give higher yield and are widely used for lipid extraction. Bacterial lipid extraction and recovery for biodiesel production are new and developing research.

### Transesterification

**Fig. 4:**
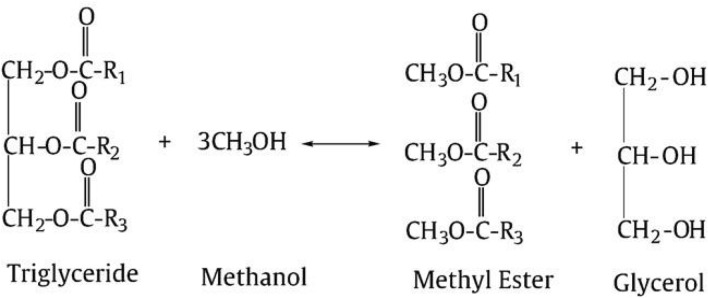
Transesterification reaction

Chemically transesterification is also called alcoholysis or esterification of triglycerides. Figure 4 shows the transesterification reaction in the presence of alcohol and a catalyst (alkali or acid or enzymes). In this reaction, triglycerides are converted into fatty acid methyl ester (FAME), with glycerol as a by-product (Ferrero et al. 2020). Some other methods are also used to produce biodiesel, such as thermal cracking or pyrolysis. Still, this process is challenging to operate and produces by-products with no commercial value. The different alcohols used for transesterification processing, including methanol, ethanol, isopropanol, and butanol, but because of low cost and availability, mostly methanol is considered for industrial production (Vazquez et al. 2020). There are many methods of transesterification of lipid shown in Table 4 and their respective yield. This current review evaluated three different transesterification methods including homogenous acid–base transesterification, heterogeneous acid–base transesterification, and enzymatic transesterification.

**Table 4:** Extraction method, lipid content and biodiesel yield (Bharti et al. 2014a; Goswami et al. 2017; Kumar et al. 2017a, b; Maheshwari et al. 2018)

S.N	Bacterial species	Lipid extraction method	Lipid content (% dry weight)	Type of catalyst used in transesterification	Biodiesel yield %
10	*Rhodococcus opacus*	Chloroform/ Methanol	40–80	H_2_SO_4_/Methanol	53.00
11	*Serratia* species ISTD04	Chloroform /Methanol	64	NaOH / Methanol	40.00–80.00
12	*Serratia* species *ISTD04*	Chloroform /Methanol	64	Immobilized lipase from *Pseudomonas* sp. ISTPL3	92.00
13	*Bacillus* species SS105	Chloroform /Methanol	57	NaOH/ Methanol	90.00

#### Homogenous base transesterification

Biodiesel is commonly produced using homogeneous base catalysts, such as sodium hydroxide (NaOH) or Potassium hydroxide (KOH). These commonly used catalysts in the industrial level biodiesel production due to several reasons such as required low reaction temperature and atmospheric pressure for catalyzing the reaction, high conversion can be achieved in minimal time, widely available, and economic. The base-catalyzed transesterification reaction is 4000 times faster than the acidic catalyst transesterification reaction. The feedstock and catalyst ratio is critical for biodiesel yield. Sometimes base catalysts, particularly NaOH, cause soap formation, and this soap with the products can significantly reduce the fatty acid methyl ester (FAME) yield. These affect the purification process, such as glycerol separation and water washing.

#### Homogenous acid transesterification

Liquid acid catalysts are proposed to overcome the limitations of liquid base-catalyzed Transesterification. They are widely used to catalyze acid transesterification, such as Sulfuric acid (H_2_SO_4_) and Hydrochloric acid (HCl). Acid-catalyzed transesterification is advantageous to the base-catalyzed process, because the acid catalyst is insensitive to feedstock free fatty acids (FFAs). It can catalyze esterification and Transesterification simultaneously. Acid catalysis is more efficient when more than 1% FFA is present in the oil. The acid-catalyzed system is not a popular choice for commercial applications because of the slower reaction rate, the requirement of high reaction temperature, the high molar ratio of alcohol to oil, separation difficulty of the catalyst, severe environmental and corrosion-related problems makes (Lotero et al. 2005).

#### Heterogeneous catalyst

The heterogeneous catalyst used to overcome the problem related to the homogeneous catalyst has easy recovery, reusability, and purification in the absence of water, a low corrosive character that makes it more suitable for biodiesel production (Gupta et al. 2017, 2020). There are two types; acid and base heterogeneous are available. The most commonly used solid-based heterogeneous catalysts are alkaline earth metal oxides, supported alkali metals, hydrotalcite, and basic zeolites (Li et al. 2019) and zeolite, mixed metal oxides, sulfonic acid group, polyoxometalates, and hetero polyacid are heterogeneous solid acid catalysts. Because of the slow reaction rate and side reaction, this heterogeneous solid acid catalyst was not a popular choice for researchers and was unsuitable for commercial biodiesel production. It recorded that the various heterogeneous catalyst broadly used for transesterification of soybean oil, waste cooking, sunflower oil, *Jatropha* oil (Vahid and Haghighi 2016; Mardhiah et al. 2017). Other than there not reported any work of heterogeneous catalysts for catalyzing Transesterification of bacterial lipids.

#### Enzymatic transesterification

The lipases (triacylglycerol acyl hydrolases) are serine hydrolases that catalyze triglycerides hydrolysis and give free fatty acids and glycerol at an oil–water interface. These enzymes are produced in the presence of a lipid such as oil or any other inducer, such as fatty acids, hydrolyzable esters, triacylglycerols, and glycerol. The lipases may use as free or immobilized forms. Transesterification reaction catalyzed by acid or alkali has many disadvantages, such as high energy consumption and triglycerides with high free fatty acid (FFA) content. The downstream processes, such as the recovery of glycerol, the removal of inorganic salts, and the removal of water and catalyst removal are complex and require additional costs. Enzymatic transesterification has an advantage over the conventional methods such as high yields and reactions done at low reaction temperatures with easy recovery of glycerol. For Industrial applications, microbial lipases are potent biocatalysts due to their potential to hydrolyze industrial waste materials. Lipase shows high catalytic activity in free form, but immobilized lipase was the best choice due to its high cost for production and recovery problems. At the industrial application of the process, the immobilization of lipase has become more vital, because it could be recovered quickly and used in continuous reactions. Immobilized enzymes are more stable toward temperature and chemical used in the transesterification process and are easy for handling, recovery, and recycle. Some particular microorganisms such as *Candida antartica*, *Pseudomonas fluorescens* were used as a source of lipase and lipase immobilization for biodiesel synthesis (Ali et al. 2017). Various oils source has used the substrate, such as *Jatropha curcas*, Stillingia oil, Castor oil, waste cooking oil (Khan et al. 2019). However, there is no report for bacterial lipid sources. Despite many advantages, biodiesel production has not been well developed commercially using enzymatic transesterification.

#### Separation, purification, and characterization of biodiesel

The trans esterified reaction mixture contains biodiesel, glycerol, and un-reacted methanol. After the completion of transesterification, the reaction mixture was allowed to settle. The mixture has been separated into two layers according to the gravity of the present components, the upper biodiesel layers (with water and methanol) and the lower glycerol layer. The lower glycerol layer is separated manually from the upper biodiesel layer. After the separation and purification, the biodiesel layer was washed with water, and excess methanol (volatile compound) was removed by a rotary evaporator (Bharti et al. 2014a). Formed biodiesel can characterize the compounds present in biodiesel using spectrophotometry methods, such as Fourier Transform Infrared (FTIR) Spectroscopy and Gas chromatography–mass spectrometry (Kumar et al. 2015; Goswami et al. 2017).

## Biotechnological approach

### Genetic engineering for lipid enhancement

**Fig. 5:**
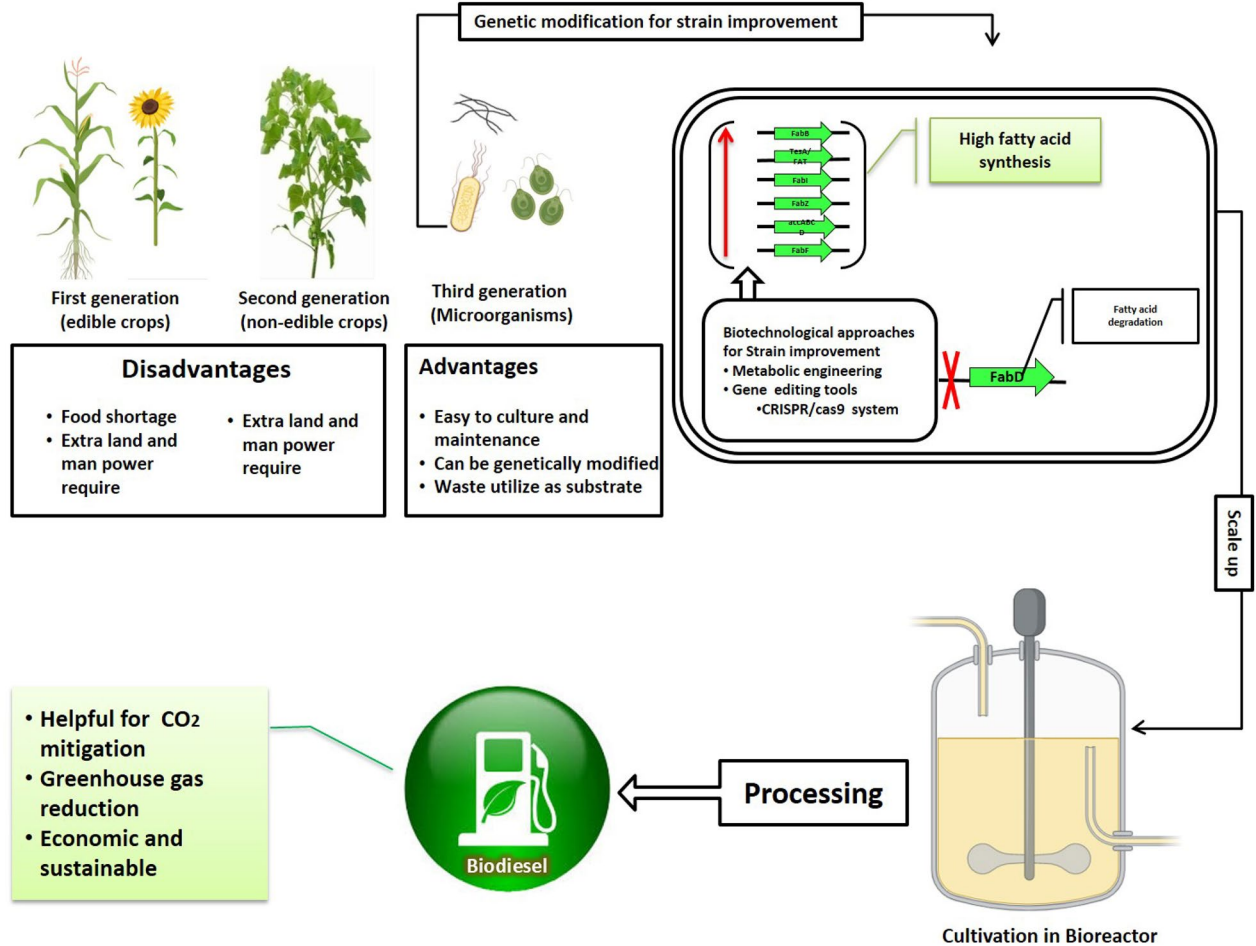
Generations of biodiesel, and future possibilities of biodiesel production from oleaginous bacteria by genetic modifications

Genetic engineering is the successful gene modification strategy for the enhancement of various metabolites compounds, such as starch, lipid, polymers and secondary metabolites (Chong et al. 2021). Multiple experiments on plants and microorganisms gene levels for targeted genetic modification (Bao et al., 2018; Qi et al. 2020; Adegboye et al. 2021). Different genes controls biochemical pathways of lipid biosynthesis and metabolism in oleaginous bacteria are displayed in Table 2. In genetic engineering strategy, these genes are either up-regulated or down-regulated for enhancing lipid synthesis, as shown in Fig. 5 and Table 5. Recently, the CRISPR/Cas9 systems were reported as a better gene-editing tool in microorganisms for biofuel production (Banerjee et al. 2017; Cho et al. 2018). With the help of CRISPR/Cas9 system desired gene can be activated or knockout by CRISPR activation (CRISPRa) and CRISPR interference (CRISPRi). CRISPR/ Cas9 systems are widely reported for producing the best strain in several bacterial genera, such as *Escherichia coli, Bacillus, Clostridium, Pseudomonas, Staphylococcus, Lactobacillus,* and *Streptomyces* (Hoang et al. 2018). Genomics, proteomics, and advanced computational technologies provide the way to screen and improve oleaginous bacterial strains, which are further used for industrial biodiesel production.

**Table 5:** List of various modified strain and their respective target gene or enzyme (Liang et al. 2013; Pfleger et al. 2015; Banerjee et al. 2017; Cho et al. 2018; Nie et al. 2022)

S.N	Bacterial Species	Gene name	Yield
14	*E. coli*	accA-D (ACC), tesA (thioesterase I)	6X fatty acid synthesis
15	*E. coli*	ACC	3 X lipid content
16	*E. coli*	*E. coli*	+ fatty acid synthesis
17	*E. coli*	FAT	> 0.2 g/L fatty acid content
18	*E. coli*	Antisense PEPC	+ 46.9% lipid content
19	*E. coli*	ACC, thioesterase, D fadD (acyl-CoA synthetase)	20 X fatty acid content

### Metabolic engineering strategies

Different biotechnological approaches such as genetic and metabolic engineering have been used to produce the desired product from various sources. Gene regulation mechanisms for fatty acid synthesis in bacteria are better known than others. Metabolic engineering is the new technique in which genetic engineering is used to modify microorganism metabolism. The primary strategy involved in metabolic engineering is optimizing existing biochemical pathways or introducing the components (Liang et al. 2013; Nie et al. 2022). This approach helped make bacterial strains that show maximum lipid accumulation capacity, which can be utilized as a lipid source for biodiesel production (Pfleger et al. 2015). Some metabolic engineering strategies are used for enhancing lipid synthesis in bacteria. Metabolically engineered *E. coli* could produce fatty acid esters using renewable carbon sources in fed-batch fermentation mode. There are the following strategies used in metabolic engineering.

#### Blocking the competing pathways

The enhanced lipid accumulation metabolism in bacteria, the undesired pathways blocked by deleting all the genes that encode enzymes for these pathways. β oxidation is one such central metabolic pathway that consumes acetyl-CoA and competes with acetyl-CoA synthesis. Many research reports enhance product formation by blocking β oxidation pathways (Pfleger et al. 2015). The deletion of acetate formation and other fermentation pathways in bacteria positively impacts acetyl-CoA levels and products made from acetyl-CoA. The transcriptional control of *gltA*can flux in the TCA cycle in *E. coli* leads to threefold increased isobutanol and acetyl-CoA-derived biofuels production.

#### Deregulation of the fatty acid biosynthesis pathways

According to Pfleger et al. (2015), the elimination of the by-pass regulation strategy also gives successful results for the increasing production of oleochemicals in *E. coli*. Acetyl-CoA synthase (ACS) catalyzes the conversion of acetate into acetyl-CoA in *E. coli*. Increased ACS level presumably enhanced the activation of acetate to acetyl CoA, increasing fatty acid synthesis, over-expressing the ACS gene in *E. coli* leads to ninefold increased activity of ACS, which significantly increases assimilation of acetate from the medium and can contribute to enhanced lipid biosynthesis. Similarly, acyl-ACP-thioesterase (FAT) FATs, also called TesA, are a group of enzymes that hydrolyze acyl-ACPs and form the free fatty acids and ACP, therefore, reducing the formation of long-chain acyl-ACP. It reported that the FAT gene introduction in *E.coli* can produce enhanced free fatty acids expresses medium-chain FAT (from *Umbellularia californica*) enzyme in *E. coli* (deficient in fatty acid degradation) and found the minor accumulation of medium-chain fatty acids. They concluded that acyl-ACP intermediates might act as feedback inhibitors for fatty acid synthesis. Depletion of the regulatory signal removes this feedback inhibition and increases fatty acid accumulation.

#### Enzyme expression regulation in lipid metabolism

The main objective of metabolic engineering is to balance gene expressions that provide sufficient enzymatic activity and avoid excess protein synthesis. Some biological approaches have been implied for balancing gene expressions, such as variation of DNA copy number, induction, promoter strength, and translation initiation rate (Pfleger et al. 2015). Zhang et al. (2021) engineered the FadR-responsive promoters for acyl-CoA levels up- or down-regulate expression in response to acyl-CoA levels when this promoter is used to regulate the expression of acyl-CoA synthetase, ethanol synthesis, and wax-ester synthetase. Its leads to threefold increased biodiesel production. Similar results found by Xu et al. (2011), when using promoters engineered to respond to FapR (the dynamic regulatory protein that responds to malonyl-CoA levels), were used and found a threefold increase in free radicals fatty acid.

## The advantage of biodiesel as diesel fuel

### Availability of biodiesel

The biodiesel domestically can be produced using renewable oilseed crops such as soybean, rapeseed, and sunflower, non-edible oil sources such as castor oil (Vazquez et al. 2020), waste food materials, wastewater, microbial oils sources (single cell oil from fungi, bacteria, and microalgae) (Wahlen et al. 2013; Zhang et al. 2020a, b). Handling, transporting, and storing biodiesel is much easier and more cost-effective than petro diesel. Biodiesel is safe to handle and transport, because it has a high flash point than petroleum diesel fuel. Biodiesel is used alone or in blended forms (mixed in any ratio with petroleum diesel fuel). Primarily B20 and B10 blends are used. B20 20% biodiesel is blended with 80% petroleum diesel, and B10 10% biodiesel has blended with 90% petroleum diesel.

### The higher combustion efficiency

Combustion efficiency is how efficiently a device or engine consumes fuel. Oxygen content in biofuel improves combustion efficiency; it increases the homogeneity of oxygen with the energy during combustion (Mishra and Goswami 2017). Depending on the production source, biodiesel contains oxygen, therefore, having higher combustion efficiency. Biodiesel also has higher heating values (HHVs), and biodiesel is more lubricating than petroleum diesel fuel; therefore, biodiesel can make longer life of diesel engines.

### Lower emissions property of biodiesel

Combustion of biodiesel reduces 90% unburned hydrocarbons (HC) and 75–90% polycyclic aromatic hydrocarbons (PAHs), particulates, and carbon monoxide than petroleum diesel fuel. Biodiesel contains a tiny amount of nitrogen as compared with petrodiesel. The N_2_O reduction was dependent on initial N_2_O concentration in the feedstock and slightly dependent upon temperature. Biodiesel also contains a trace amount of sulfur, so SO_2_ emissions reduce by biodiesel (Mishra and Goswami 2017; Sheth et al. 2020). Biodiesel in conventional diesel engines, the emission of unburned HC, carbon monoxide, sulfates, PAHs, CO_2_, nitrated PAHs, ozone-forming HC, and PM can be substantially reduced (Kaya and Kokkulunk 2020). Biodiesel blends are preferred in engines, because they can decrease power, torque, and NOx emissions (a contributing factor in the formation of smog and ozone) (Dahman et al. 2019). Recently, various additives have been added to biodiesel for improving engine performance. Shadid et al. (2020) reported that using nanocatalyst containing fuel blends can reduce the emission of CO_2_, HC, and NOx.

### Biodegradability of biodiesel

Biodiesel has degraded about four times faster than petrodiesel. Therefore, because biodiesel contains oxygen, it shows poor oxidative stability compared to petrodiesel. Biodiesel contains unsaturated and polyunsaturated FAME that are more susceptible to oxidation. This biodiesel quality makes it very advantageous for the environment (Reddy et al. 2020). After 28 days, biodiesel fuels have 77–89% biodegradability, and diesel fuel was only 18% biodegradability (Dahman et al. 2019).

## Biofuels policies and Future prospect for biodiesel

Energy is an essential component of the economic development of the county. The growing population and their enhanced living standards generate continuous energy demands, and most of the energy demands have been fulfilled by conventional fossil fuels. Fossil fuels generate a direct and indirect impact on health, the environment, and the economy. Renewable energy, such as biofuel, is the best alternative energy source to reduce the dependency on fossil fuels (Gautam et al. 2019; Ziolkowska 2020). Biofuel is a sustainable fuel alternative, and its production is encouraged by both developed and developing countries (Demirbas et al. 2008; Araujo et al. 2017). Therefore, the development and implementation of biofuel require a biobased economy and policies. These policies create a chain mechanism from production to supply of biofuel. Around 65 countries are made their formal biofuel policies by 2018. Therefore, biofuel use increases (Acharya and Perez-Pena 2020). The United States, Brazil produced nearly 90% of the ethanol, and the European Union countries and the remaining 10% produced by China, Canada, India, Thailand, and Argentina by 2018 (Acharya and Perez-Pena 2020). Biofuel policies adopted by different countries have similar objectives, such as concern for environmental impact, feedstock availability, rural development, promoting industrialization, enhanced blending ratio with fossil fuel, and reducing the dependency on fossil fuel (Araujo et al. 2017; Acharya and Perez-Pena 2020). Government policies also play a central role in promoting and nurturing research, innovation, and implementation and encouraging the biofuel's market (Sinha et al. 2019).

Oleaginous microbes are the lipid source for biodiesel production for the upcoming generation. These oleaginous bacteria are cultivated on various agricultural residues and waste substrates. Sewage sludge consists of untreated municipal liquid waste, containing about 99.9% of water, while the remaining content might be organic or inorganic. This sludge was prone to the growth of different harmful microbes and caused land, air, and water pollution, negatively impacting the environment (Karn and Kumar 2019). Therefore, utilizing this waste as a substrate by oleaginous bacteria and producing biodiesel is effective. Thus it has promoted waste management, such as minimization, recycling and disposal. There are vast research possibilities of biodiesel production from oleaginous bacteria as lipid sources. In the future, significant challenges related to bacterial biodiesel would be to search and develop bacterial strains with a high lipid profile. Not all bacterial strains accumulate sufficient lipid at the cultivation stage; therefore, new advanced metabolic engineering strategies can opt for bacterial strain development and optimizing cultivations condition for both small scale and industrial levels. The path of researches opportunities has opened on the following perspectives in the future:

1. Oleaginous bacteria strain selection—various techniques will search for the best strain with high lipid accumulation. Biotechnological approaches will apply for the development of the best bacterial strains.
2. Alternative substrate selection and its utilization for the cultivation of bacteria for biodiesel production make the overall process very cost-effective and positively impact the environment.
3. Physiological cultivation conditions of microbes are necessary to understand for high yielding of by-products.
4. It is making an effective lipid extraction technique that utilizes a solvent system at a low cost. Transesterification is an essential step of biodiesel production. Therefore, knowing the best catalyst for bacterial lipid transesterification will be needed and give a good FAME profile.
5. The various catalysts are available, but very few are reported for bacterial lipid transesterification. Therefore, there will be an open possibility to research the novel catalyst for bacterial lipid.
6. Separation and purification are needed to finish the overall process with an efficient solvent system that gives high-quality biodiesel with minimum undesired by-products formation. FAME profile analysis ensures that the bacterial biodiesel that fits ASTM (American Society for Testing and Materials) standards will suit engine performance.
7. Finally, we require a new and advanced policy in the future, which can promote biodiesel production from oleaginous bacteria and other microorganisms.

## Conclusions

Increasing population, industrialization, and environmental pollution consequences generate high energy demand that is suitable or friendly for our environment. Biofuels are an alternative, renewable energy source that is more suitable for the environment. It is a third-generation biofuel that reduces the dependency on fossil fuels for energy production. Bacteria are an excellent prominent resource for an upcoming generation as a suitable feedstock for lipids sources. An oleaginous bacterium has tremendous benefits. Its feedstock has many advantages such as a short life span, a high growth rate, and is easy to cultivate on different substrates. At the cellular level, metabolically and genetically modification ease. Providing various nutrients and the C/N ratio in the medium will also change the lipid composition and total biomass yield. Bacteria can metabolize various carbohydrates, such as residual sugar from food, and grow multiple waste organic materials, such as wastewater, food waste, dairy wastewater, and sewage sludge. Biodiesel production by microorganisms can also be helpful for waste management and sustainable development. Various lignocellulosic agricultural wastes can use as a substrate for bacteria cultivation. Microbial oil production from oleaginous microorganisms uses different nutrient-rich wastewaters, and this microbial oil used for biodiesel production has great potential for industrial application. It provides an advantage for wastewater treatment by removing COD and nutrients from the bacteria's wastewaters and producing renewable oil for biodiesel production. Some bacteria species give us dual benefits, such as microalgae; they utilize CO_2_ called CO_2_ sequestrating bacteria. They also accumulate a significant amount of lipid. Every step involved in biodiesel production affects biodiesel's overall quantity and quality. Therefore, we need to use the best-optimized method for each step. Future research needs to implement lab-based bacterial biodiesel production methods to make industrial and commercialized levels.

## Data Availability

Not applicable.
